# Masticatory Adaptation to Occlusal Changes

**DOI:** 10.3389/fphys.2020.00263

**Published:** 2020-04-03

**Authors:** Pierre Bourdiol, Martine Hennequin, Marie-Agnes Peyron, Alain Woda

**Affiliations:** ^1^CROC EA 4847, Faculty of Dentistry, Université Clermont Auvergne, Clermont-Ferrand, France; ^2^CHU Clermont-Ferrand, Clermont-Ferrand, France; ^3^Human Nutrition Unit, Institut National de la Recherche Agronomique, Paris, France

**Keywords:** masticatory adaptation, occlusion, dental wear, masticatory test, disability, tooth loss

## Abstract

This review deals with the frequent wide variability of masticatory capacity/incapacity. Neither researchers nor clinicians have taken sufficient account of this variability despite its implications for nutrition. Mastication in normal healthy oral conditions is first described, followed by a short presentation of the mechanisms of masticatory adaptation in the nervous system. Capacity, incapacity, and successful compensatory adaptation of mastication are then defined, along with the different methods used for their evaluation. Examples of adaptation needs are given, such as those concomitant with dental wear or occlusal changes. Finally, given its vital importance for deeply impaired mastication/deglutition function, the impact of masticatory adaptation processes on nutrition is examined.

## Physiology of Normal Mastication

Ingestion starts with the choice and selection of a food and ends with its passage through the upper esophageal sphincter during deglutition. A first model, based on common observation, considers that ingestion is controlled at three checkpoints, each of which can cancel ingestion. The first checkpoint is food selection before ingestion. The second consists of sensory cues from food. The third is deglutition. A second model is the four-step sequence described by Hiiemae and Palmer (1999) for the fate of solid or semi-solid foods in the mouth.

**FIGURE 1:**
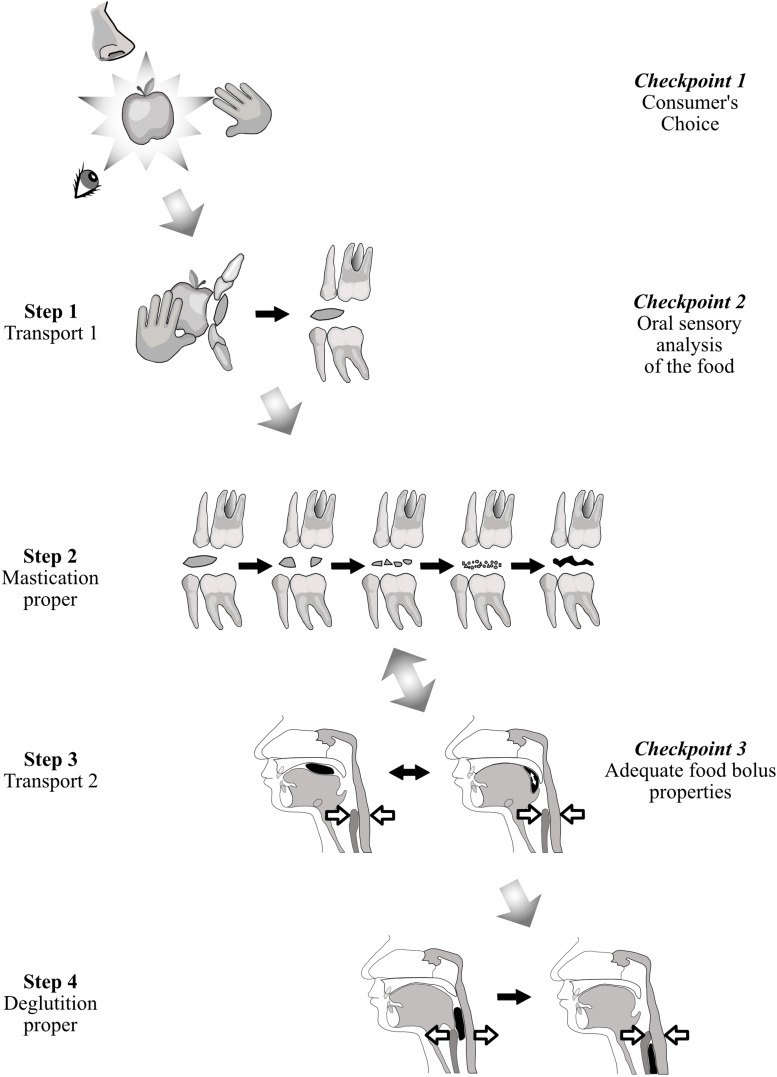
Solid food from identification to first bite and mastication/deglutition. While it is transported, the solid food is in turn examined, bitten, masticated and finally swallowed. This process can be considered from the point of view of either food transport (Hiiemae and Palmer, 1999) (first column on the left), or controls at three checkpoints (last column on the right).

Figure 1 relates these two models. At the first checkpoint, individuals express their personal choice when they shop for food, and when they accept or decline food they are proffered. After selection, solid or semi-solid foods are portioned into mouthfuls, typically with the front teeth or with eating utensils. This portion is then transported from the front teeth to the molars (Hiiemae and Palmer’s Stage I transport). During this phase, each mouthful is analyzed by taste, retro-nasal olfaction and oral receptors of the somatosensory system. If this second checkpoint is successfully crossed, then (i) the central pattern generators of mastication located in the cerebral cortex and in the brainstem are activated, and (ii) the required physiological responses are anticipated to prepare digestion of the food in the digestive tract post-esophagus (cephalic phase). This cephalic phase also occurs during mastication proper (Hiiemae and Palmer’s second step), when the food is transformed into a food bolus by the actions of the teeth through the exertion of lingual, facial and masticatory muscles and with the aid of saliva. In the third step, Hiiemae and Palmer’s Stage II transport, the chewed food moved rearward along the oropharyngeal tongue surface, crosses the fauces isthmus and collects in pharyngo-epiglottic folds. The fourth step, acting as a third checkpoint, is deglutition proper, with opening of the upper esophageal sphincter. Steps 2 and 3 often occur simultaneously, the bolus being moved cyclically upward and forward on the tongue surface, returning through the fauces isthmus into the oral cavity while the mastication proper is taking place. After passing through this third checkpoint, chewed food is irreversibly delivered to the gastro-intestinal tract, from which it can then be expelled only by vomiting. To cross this last checkpoint, a deglutition center’s “go-ahead” is needed, signaling that the bolus is sufficiently well prepared to be easily and safely swallowed. Many studies have shown that the particle size of the bolus just before swallowing is a determining factor (van der Bilt et al., 1993; Jalabert-Malbos et al., 2007; N’Gom et al., 2007). Particle size below a ceiling is one necessary condition for swallowing to be triggered. Particle size acts as a cofactor with saliva and food juice to reach the required rheological properties for the food bolus. To be safely swallowed, the bolus must possess certain physical and rheological properties. It must be slippery, cohesive and plastic (Prinz and Lucas, 1995). Plasticity allows the deformation of the bolus during its passage through the digestive tract. Slipperiness helps it slide along the mucous membranes and down the narrow alimentary canal to the stomach. Cohesiveness means that the bolus must behave like a unit (Hutchings and Lillford, 1988; Palmer and Hiiemae, 2003). This is essential to avoid aspiration, which can happen if food particles disperse and enter the airways when the bolus crosses the aerodigestive junction. It is noteworthy that subjects neither stop chewing nor trigger deglutition when the required mean particle size has been reached (Peyron et al., 2011). They masticate longer, increasing the number of cycles, to obtain all the necessary rheological conditions described above (Prinz and Lucas, 1995; Seo et al., 2007; Mishellany-Dutour et al., 2011) by mixing the solid particles with saliva and juice expelled from the crushed food. This probably accounts for the weak correlation between number of cycles and pre-swallow median particle size (van der Bilt et al., 1993; Fontijn-Tekamp et al., 2000; Mishellany-Dutour et al., 2011).

Hiiemae and Palmer’s Step 2 consists of a chewing sequence comprising a variable number of masticatory cycles (Fontijn-Tekamp et al., 2004; Peyron et al., 2004; Lund and Kolta, 2005). Food placed in the mouth acts as a stimulus for sensory receptors. This stimulation by food occurs both at the start and then throughout the masticatory sequence as the food is progressively transformed. Through this continuous sensory input, the mastication generating centers adjust several parameters, such as number of cycles before swallowing, muscular force exerted, and shape of the mandibular movement (see Table 1 in Woda et al., 2006). These parameters are adjusted to (i) continuously adapt to food properties inside the mouth and (ii) obtain required final food bolus properties. The need to reach a final state of the boluses, which is similar for all healthy individuals, is met using all the available means. This drive for adaptation produces strong inter-individual differences in the masticatory sequence. If the right bolus properties cannot be attained, subjects either swallow insufficiently comminuted foods, or avoid eating the food, which they deem difficult to chew. We note that only in experimental conditions can refusal of food types be assessed (Hennequin et al., 2015; Depeyre et al., 2019).

## Mechanisms of Mastication Adaptation

Variation of the mastication parameters allows adaptation to several types of changes deriving from either the environment (extrinsic) or the individual (intrinsic). Four adaptation/variability situations can be distinguished: (i) the cycles of mastication vary during a single sequence to adapt to the changes in the food during its transformation into a food bolus, (ii) the programming of mastication varies to adapt to food types, (iii) mastication undergoes slow reprogramming to adapt to evolving conditions such as orthodontic movements, dental wear or aging, and (iv) mastication must also adapt to discontinuities such as tooth loss or prosthodontic occlusal rehabilitation. There is neuroscientific evidence for these different types of adaptation/variability.

All the forms taken by mastication adaptation have a common foundation, namely the rhythmicity and coordination of masticatory muscles, tongue, lips, and cheeks, which depend on a central pattern generator located in the brainstem (Lund and Kolta, 2005). Central to this rhythmicity is the presence of neurons with rhythmogenic properties in one area of the central pattern generator: the rostro-dorsal part of the trigeminal sensory complex. These neurons have intrinsic calcium- and voltage-dependent bursting abilities. The integration of peripheral and cortical inputs by these neurons could partly explain the first type of variability/adaptation, i.e., the instantaneous variations in cycles occurring while mastication proper is progressively transforming the food into a food bolus (see review in Morquette et al., 2012). Chewing patterns can also change suddenly in response to events caused during mastication, such as pain (temporo-mandibular joint, dental, or mucosal), or foods sticking to gums or teeth. Periodontal, muscle spindle, mucosal and other peripheral receptors all act to adapt to hardness and other rheological modifications of the food during mastication and to protect the apparatus from unexpected perturbations, which may be of a nociceptive nature. Responses reach the brainstem central pattern generator directly, but also through cortical areas, which form part of a feedback loop. That the cortex plays a role is indicated by the fact that if the masticatory area is suppressed, mastication, though still possible, becomes awkward and slowed (see Lund and Kolta, 2005; Avivi-Arber and Sessle, 2018; Kumar et al., 2018).

The second type of adaptation, i.e., adaptation of mastication to each food type, is probably programmed before the mastication sequence starts. Cortical inputs seem essential to activate, directly or indirectly, the brainstem central pattern generator with the program appropriate to a given type of food, as suggested by the representation, at specific locations within the cortex, of maps of the various patterns of mastication (Lin et al., 1998; Lund and Kolta, 2005). This pre-ingestive programming can also be inferred from the fact that cognitive, visual and olfactory information about food type reaches the cortex before the first bite, while somato-sensory, retronasal olfactory and taste information is collected during the initial transport of food in the mouth and before the beginning of mastication proper (see Lund and Kolta, 2005, 2006; Kumar et al., 2018).

Cortical plasticity seems to underlie the adaptation of mastication accompanying evolving conditions such as orthodontic movements, dental wear or aging, and more sudden events such as tooth loss or chance injuries of the orofacial area (see recent and complete reviews in Avivi-Arber and Sessle, 2018; Kumar et al., 2018). The plasticity of the central nervous system has long been known, but it is only recently that the role of this plasticity has been studied in detail for cortical areas devoted to orofacial function in the primary somato-sensory cortex and in the primary motor cortex. Among other techniques and experimental designs, intracortical micro-stimulation allows the observation of adaptive changes in some oral functional tasks in rats. Functional magnetic resonance imaging or transcranial magnetic stimulation have been used in humans for related purposes. Reorganization of sensory and motor representation and/or modification of the excitability of the orofacial cortical region have been shown following events such as occlusal grinding, tooth extraction and nerve injury (Avivi-Arber et al., 2011), but also after gradual changes such as orthodontic movements (Sood et al., 2015). This adaptive plasticity can be positive by helping mastication adapt to the new conditions, thereby limiting the dysfunction, but may also lead to new maladaptive motor habits, either postural or kinetic. Cortical plasticity is also involved when a subject is learning a new task or receiving a new complete denture. Adaptation to a new complete denture correlated with plasticity of cortical motor area in a time-dependent manner (Luraschi et al., 2013). Neuroplasticity induced by tooth extraction can be reversed by replacement with an implant-supported crown, although it does not return to its initial state before extraction (Avivi-Arber et al., 2015). As pointed out by Avivi-Arber and Sessle (2018) “Such information has clinical significance as cortical changes may underlie the mechanisms by which humans adapt (or not) to intra-oral manipulation.” Finally, these new scientific data reinforce the old insufficiently applied clinical concept that training of oral motor tasks or relearning of initial masticatory praxis after occlusal rehabilitation would help users adapt to their new dental prosthesis.

The swallowing function also adapts to the food bolus. The activation of the relevant oropharyngeal muscles occurs in an invariable order, but the intensity of muscle activities and the overall temporal aspects of muscle events are influenced by bolus characteristics such as volume (Kahrilas and Logemann, 1993; Lazarus et al., 1993), viscosity (Lazarus et al., 1993; Smith et al., 2006) and taste (Ding et al., 2003). In normal conditions, swallows frequently occur intermittently during the chewing sequence until the final food bolus is swallowed (Hiiemae et al., 1996; Okada et al., 2007).

## Capacity with or without Compensatory Adaptation, Incapacity

**FIGURE 2:**
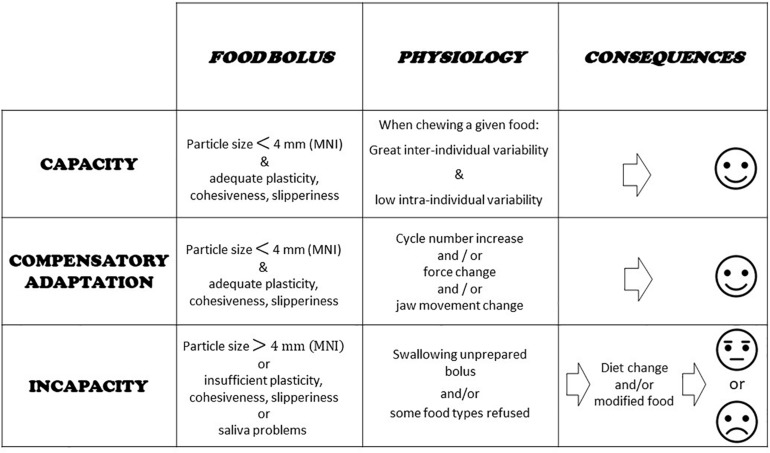
Capacity, compensatory adaptation, and incapacity. Evaluation of food boluses is proposed through particle size measurement and a masticatory normative indicator (MNI) made with raw carrot. Main physiological characteristics at work during adaptation are indicated. Basic consequences are shown.

The main question when evaluating the masticatory function in a subject or group of subjects is whether this function achieves its purpose. In other words, whether subjects are able to make an acceptable food bolus or whether the food texture has to be changed. This tells us how well the mastication centers adapt to the characteristics of both the food and the eater. In some individuals, the mastication process may lie close to the border of normal healthy functioning. In these situations, adaptation may be difficult and costly, though still possible. It may be impossible in other situations. These three conditions: totally healthy, moderately impaired and totally impaired, determine masticatory capacity, compensatory adaptation, and masticatory incapacity (Feldman et al., 1980; Woda et al., 2010, 2011). Capacity means that chewing is perfectly achieved, and the masticatory capacity is fully intact. The food bolus meets all the requirements for deglutition as established with an indicator of normal food bolus such as MNI (see next section). Compensatory adaptation occurs when mastication is slightly disturbed, but the individual concerned can implement a physiological adaptation, mainly an increase in the number of cycles, that makes a normal bolus. Compensatory adaptation implies extra effort, but normal values of MNI are reached. Incapacity means the function is largely deficient, because the individual fails to make a proper food bolus or refuses it in some way. In such subjects, adaptability is overstretched; they are unable to masticate correctly as shown by the MNI value, which is above the ceiling. The subjects have to develop a strategy that enables them to feed themselves despite their incapacity. The main strategies are: changing diets, avoiding foods that are difficult to chew, and swallowing unchewed food. In all cases, negative nutritional consequences and/or excessive workload inflicted on the digestive tract are likely. The morbid outcomes of this situation are still under-researched but appear more and more probable and serious (El Osta et al., 2014). These three conditions are summarized in Figure 2. Compensatory adaptation or incapacity can be found in many settings such as craniofacial dysmorphia, neurological diseases, traumatic or surgical sequelae, temporomandibular disorders and other conditions leading to occlusal changes including partial or complete edentulousness.

Epidemiological evaluation of the prevalence of mastication incapacity in the general population is incomplete. Prevalence of mastication incapacity is almost totally lacking for persons with neuromotor and cognitive disorders such as Parkinson disease, stroke, congenital or acquired brain damage and other neurological disorders. It can, however, be inferred to be high since these conditions are common and concern both young and aged individuals. In addition, epidemiology of mastication inability in cognitive and neuromotor disorders has been hidden by the emphasis placed on dysphagia which showed very high values, up to 80% of stroke patients and 81% of Parkinson disease patients (Takizawa et al., 2016). It is worth noting that the major role of mastication incapacity in the dysphagia states was never considered. Epidemiology of mastication deficiency in the elderly is better known. However, the respective role of neurological disorders and of edentulousness in this aging population is difficult to determine because, in these studies, elderly with cognitive and neurological disorders had been either discarded (Cavalcante et al., 2019) or not reported.

Fifty-three percents of a Brazilian population made up of 5,124 elderly individuals (aged 65–74; 59.2% wearing complete denture) declared they had poor mastication (Dias-da-Costa et al., 2010). In a group of 3,134 Japanese community dwelling elderly persons with a median age of 71, 20.7% could not eat one of the five test foods proposed (Okamoto et al., 2019). As concluded by the authors the subjects’ difficulties were strongly correlated with the number of residual natural teeth. Indeed, a large proportion of them (786, 25%) had less than 10 teeth with a median of two teeth remaining. A similar value (21%) was reported in a cohort of Australian elderly persons aged over 78 years, who could not eat hard foods and reported both discomfort and meal interruption while eating. Only 14,6% wore complete denture but partial edentulousness was widespread. There was a 2.3 times greater likelihood that those with fewer than 21 natural teeth were not able to eat hard foods (Wright et al., 2019). These results are in line with the well-known fact that complete denture patients have mastication difficulties (van der Bilt, 2011; Peyron et al., 2017). In a cross-sectional study conducted in north-east Brazil with a random sample of 532 participants aged 20–59 years, the prevalence of declared chewing difficulties was 30.5% (Cavalcante et al., 2019) with 8.7% having less than nine remaining natural teeth.

## Tools for Mastication Evaluation

Maintaining or rehabilitating masticatory function is a dentist’s main goal. Achieving it often relies on reconstructing normal anatomy using various criteria, such as bilateral symmetry. However, the underlying assumption that good morphology implies good physiology is somewhat specious. Given the importance of masticatory function, it is essential to be able to assess and quantify it simply and reproducibly, like any other function. Various methods are available to practitioners.

### Number of Posterior Functional Units

A posterior functional unit (PFU) can be defined as a pair of antagonist posterior natural (or fixed prosthodontic) teeth with at least one contact during chewing. A fully dentate subject has eight PFUs in the form of premolar-molar contacts between maxillary and mandibular natural teeth. The number of PFUs may reach ten if third molars are included. Many malocclusions are morphologically based, although they may allow perfect functioning. For this reason, PFUs must be recorded *in vivo* using a functional test with interposed articulating paper bitten during simulated chewing movements (Hennequin et al., 2015). The role of opposing teeth in mastication is straightforward. Many studies have shown that mastication performance is reduced by loss of posterior teeth, since these are the active tools in food comminution (Helkimo et al., 1978; Feldman et al., 1980; Kohyama et al., 2003; van der Bilt, 2011; Hennequin et al., 2015). The utility of recording PFUs is further illustrated by El Osta’s study conducted in 200 aged individuals characterized by their nutritional status with the Mini-Nutritional Assessment questionnaire. Variations in PFU number largely explained nutritional status (El Osta et al., 2014). The precision of this PFU method has been criticized (Yurkstas and Manly, 1949). This may be an issue for basic experimental research, but in clinical experiments, the method is simple and useful.

### Self-Administered Questionnaires

Masticatory function can be subjectively evaluated by self-administered questionnaires. Such questionnaires are often used in epidemiological surveys. They can also be useful when evaluating a change induced by a therapeutic intervention in a specific population. For example, a questionnaire of this type usefully evaluated the benefit for edentulous populations of wearing implants (Feine and Lund, 2006). However, their use is generally restricted to particular pathological and/or experimental situations, and they have no general validity for the whole population (Allison and Hennequin, 2000; Feine and Lund, 2006). In addition, these questionnaires tend to give an overly favorable result for chewing, and their results show little correlation with those of the objective evaluation methods (Slagter et al., 1992; de Lucena et al., 2011; Cusson et al., 2015). They may be useful when considering the overall oral function including swallowing, salivation and other oral functions. The Oral Health Impact Profile (OHIP), the Geriatric Oral Health Assessment Index (GOHAI), and the International Classification of Functioning, Disability and Health (ICF) are examples of such frequently used questionnaires mostly related to oral quality of life or classification purposes (Atchinson and Dolan, 1990; Slade and Spencer, 1994; Faulks et al., 2013; Dougall et al., 2018).

### Particle Size of the Food Bolus

The purpose of chewing is to reduce food to small particles and mix these with saliva. The size of the particles forming the food bolus just before swallowing is one deglutition triggering factor. Accordingly, measuring food bolus particle size is considered the “gold standard” for objective mastication evaluation. Test foods are most often carrots or peanuts as natural foods, and elastomeric compounds (Optosyl^®^ or Optocal^®^). The median particle size (d50) is determined. The d50 corresponds to the mesh of a sieve that lets through one half of the mass of the particles and retains the other half. A *chewing test*, or chewing efficiency test, is based on determining the d50 at a predetermined number of cycles (usually 10 or 20 cycles). It gives an indication of chewing efficiency. We note the possibility of low efficiency in subjects with a healthy mastication made of long sequences composed of low power cycles. The *chewing test* must be differentiated from the *mastication test*, which gives a direct indication about the mastication capacity (Bonnet et al., 2019). In the mastication test, the d50 is evaluated from a bolus collected just before deglutition. A cut-off value for normality of 4 mm has been demonstrated with raw carrots (Woda et al., 2010) or artificial food (Witter et al., 2013). This value corresponds to the Masticatory Normative Indicator (MNI) beyond which the analyzed subject can adapt no further, the upper limit of capacitive adaptation being reached. Raw carrot was chosen as edible test food because it is hard to chew, and best reveals a subject’s deficiency or difficulty in adapting. It may be refused by patients, clear evidence of deficiency (Depeyre et al., 2019). However, it may hide partial improvements during a therapeutic trial. Clearly, because of their relative complexity, particle size evaluation and other measurements of the rheological properties of the pre-swallowing bolus belong to experimental research.

### Physiologic Methods of Evaluation

Chewing can be evaluated by electromyography, kinematics, force sensors or video (Hennequin et al., 2005; Nicolas et al., 2007). Muscular strength, amplitude and trajectory of the masticatory cycles of the mandible, duration of a mastication cycle, and frequency of chewing cycles can thus be evaluated. Owing to their characteristically complex nature, these methods are used in an experimental context only. Many experiments have studied the role of rheological properties of food, hardness, plasticity, viscosity or elasticity, to observe the adaptation of mastication to these extrinsic stimuli. One related question concerns the choice of the test food. Natural foods are a first choice because subjects can accept and swallow them easily. However, for better control of the rheological properties, several groups of experimenters have introduced model foods whose homogeneous and reproducible structure, controlled physical properties, and controlled size and shape, make it possible to observe the impact of a variation in hardness on the masticatory parameters (Nicolas et al., 2007; van der Bilt, 2011). The first model foods were made from elastomers. More recently, edible model foods were introduced. They were made of gelatin, which offers the possibility of graduating hardness independently of the other rheological properties (Peyron et al., 2002).

### Routine Clinical Evaluation of Masticatory Function

The color-mixing test could be used routinely to evaluate masticatory function. The advantage of this chewing test is that it can be easily used in daily dental practice to determine the masticatory capabilities of a subject before and after oral rehabilitation. The colorimetric test demonstrates the ability of an individual to homogenize an initially two-color support into a single monochrome phase, and thus to form a homogeneous bolus. Different materials have been used: two-tone chewing paste, two-tone wax and two-tone silicone (van der Bilt et al., 2010b). The most promising test material is a two-color chewing-gum that is chewed by the subject for a defined number of cycles and then spat out; the mixture of the two colors is evaluated either visually by direct observation or with analysis software (Schimmel et al., 2007, 2015; van der Bilt et al., 2012; Halazonetis et al., 2013). The method was validated by comparison with the results obtained by particle size measurement (van der Bilt et al., 2010b). This evaluation can also be visualized using a 5-stage color mixing reference scale (from Stage 1: unmixed chewing-gum, to Stage 5: perfectly mixed chewing-gum when the bolus color is uniform throughout). The simplicity in use of the colorimetric test lends it potential for clinical application (Elsig et al., 2015). Another attractive proposal relies simply on visual observation of food boluses immediately after they are produced. The bolus is compared with a chart composed of photographic pictures of 5 or 10 food boluses ranging from very well prepared to unprepared (Nokubi et al., 2013; Igarashi et al., 2019).

## Adaptation of Mastication to Dental Wear

The most obvious example of slowly occurring, directly visible changes of the dental arches is dental wear, which offers an example of a gradual, reciprocal adaptation of mastication and occlusion.

### Normality of Dental Wear

Pioneer observations and more recent studies have amply shown that dental wear is normal. Dental wear is a general phenomenon found in all mammals, in every civilization, and at all ages (d’Incau et al., 2012). Its fullest extent is seen in ancient populations (d’Incau et al., 2012). It is also observed in populations who currently still have an “archaic” way of life and eat non-industrially processed natural foods (Campbell, 1925; Begg, 1954; Davies and Pedersen, 1955; Murphy, 1959b; Beyron, 1964). Examples of these populations include Australian Aboriginals, Inuit, and native North Americans. The severity and shape of the worn surfaces in ancient populations or modern ones with “archaic” ways of life are mainly related to diet, with abrasive foods, the individual’s natural environment, and food processing technology (Kaifu et al., 2003; d’Incau et al., 2012). Dental wear is also found in all individuals in populations of developed countries, where it occurs at a slower pace (Woda et al., 1987). The low level of dental wear in modern civilization is probably due to more industrially processed foods that need less forceful mastication and are less abrasive (d’Incau et al., 2012). Whatever the population observed, all the occlusal wear facets are formed during the different masticatory cycles. For the incisors, some wear facets are associated with protrusion/retrusion movements. However, at the premolar/molar level, they are associated with lateral/medial movements, creating two types of facet: working and non-working, which are equally functional (Woda et al., 1979). When they are multiple and evenly distributed, they are superimposable on the occlusal contacts during maximum intercuspation. Finally, dental wear reshapes and adapts the morphology of the dental arches. Dental wear appears as a correlate of masticatory function, since dental wear facets guide masticatory movements, which in turn are the cause of dental wear (Hildebrand, 1936; Anderson and Picton, 1957; Beyron, 1964; Pameijer et al., 1969; Woda et al., 1987; Kim et al., 2001).

### Tooth Displacement and Dental Wear

Reciprocal relationships link dental wear to several other phenomena. Loss of dental material due to dental wear results in tooth displacement to keep the continuity of contact between upper versus lower teeth and between adjacent teeth. In the vertical direction, wear of the occlusal table is compensated for by continuous active dental growth (Compagnon and Woda, 1991), without which an increase in facial vertical dimension may result (Begg, 1954; Tallgreen, 1957; Berry and Poole, 1976). Cementum apposition near the apical part of the dental root increases root length. Mesial drift of the posterior teeth ensures contact between adjacent teeth and compensates for the proximal wear. Lingual tilt of the anterior teeth accompanies the change from initial psalidodontia (with overlap) to labidodontia (end-to-end) with advanced wear (Kaifu, 1996) helping to maintain occlusal equilibrium (Kaifu et al., 2003; d’Incau et al., 2012; Garot et al., 2016).

One interesting condition is when there is an intercalated posterior tooth loss in an otherwise healthy dentition. Study of this situation showed that after their initial eruption, teeth continued to evolve in the occlusal direction during adulthood, through two mechanisms: egression, through an addition of alveolar bone, and eruption, by the tooth growing out of its socket (Murphy, 1959a; Compagnon and Woda, 1991). This latter component increases the ratio of crown to root length, the negative impact of which on biomechanical equilibrium is counterbalanced by the apposition of cementum at the apex of the root. These two components of vertical dental movement interact with different degrees of dental wear in a complex equilibrium that depends on the amplitude of masticatory or other external applied forces and physiological or pathological variations of dental arches. Several situations are possible. Weak masticatory forces lead to discrete occlusal wear, resulting in an enhanced occlusal vertical dimension due to the continuous egression of all teeth (Tallgreen, 1957). Conversely, intense masticatory forces are frequent in archaic civilizations. This may enhance egression, resulting in the preservation of the occlusal vertical dimension despite abrasion of all dental crowns as observed in Inuit populations (Berry and Poole, 1976). Lack of antagonist teeth favors both permanent egression and permanent eruption, the latter phenomenon being preeminent in inflammatory conditions.

Balance between proximal wear and movements occurs between adjacent teeth. There is a migration of all teeth forward from the third molar toward the first incisor; this phenomenon is also called mesial migration. This movement maintains contact between adjacent teeth and reduces the frequency of malocclusion and crowding of anterior teeth by decreasing tooth size. More space is thus left for the eruption of the third molar (Begg, 1954).

### Dental Wear Origin

The origin of dental wear evidences the relationship between mastication and dental wear. Several studies have shown that dental wear is due to abrasion caused by very small hard particles found in various parts of many plant species. These silicate particles are called phytolyths (Lanning and Eleuterius, 1985; Hart, 1988; Piperno, 1989). Other abrasive particles like sand grains or shed dental enamel may also serve as abrasive materials (Lewis and Dwyer-Joyce, 2005). The food itself may polish the pits and grooves made by these abrasive materials, whose mean half-life is very short, much less than 1 month (Morel et al., 1991).

### Dental Wear May Be Abnormal

Although wear is less evident in modern civilization, it sometimes takes abnormal forms that can cause pain and alter function and/or esthetics. Marked occlusal wear can be due to parafunctional habits and/or dysfunctional behavior such as bruxism. When abrasion of occlusal surfaces is excessive, physiological egression may fail to compensate for loss of tooth height and may result in reduction of the facial vertical dimension and in accentuation of mandible closure in maximal intercuspal occlusion. With such excessive abrasion, anterior occlusion progresses toward an end-to-end anterior relationship and finally to mandible protrusion or prognathism, and sometimes a deep-bite face with advancement of the chin. This loss of vertical dimension has a progressive repercussion on soft tissue profile (Begg and Kesling, 1977; Richards, 1985; Planas, 1987; Kaifu et al., 2003; Kaidonis, 2008; d’Incau et al., 2012). Conversely, insufficient wearing, often found in modern populations, may have unwanted consequences: dental wear may be hidden or hindered by therapeutic reconstruction (Anderson and Myers, 1971; Woda et al., 1987). In addition, inconspicuous or insufficient occlusal dental wear, together with the continuing vertical egression of teeth, can result in an increase in the occlusal vertical dimension (Tallgreen, 1957). This may place periodontal tissues in an unfavorable condition. Also, a general decrease in occlusal wear explains the recourse to therapeutic occlusal management of interarch contacting surfaces (Planas, 1987; d’Incau et al., 2012), i.e., occlusal equilibration, in prosthetic cases and in cases of temporo-mandibular problems (Kurita et al., 2001; Pereira et al., 2009; van der Bilt, 2011). The disappearance of proximal tooth wear favors a tendency to crowding induced by physiological mesial migration. This effect was the basis of an orthodontic technique promoting tooth reduction in material and number (Begg and Kesling, 1977; Planas, 1987; Kaifu et al., 2003). It has also been suggested that less dental wear gives free rein to functional disorders encountered in modern populations such as temporo-mandibular problems (van der Bilt, 2011).

### Dental Wear and Masticatory Capacity/Efficiency

Dental wear increases the interarch contact area and participates actively in interarch adjustment. This in turn increases the efficiency of mastication (Yurkstas, 1965). Inversely, the decrease in functional occlusal surface may reflect a decrease in masticatory efficiency (Yurkstas and Manly, 1949; Julien et al., 1996). Occlusal contact area of the postcanine teeth is correlated with the small median particle size obtained at the end of a mastication sequence (Wilding, 1993; Julien et al., 1996; English et al., 2002; Lepley et al., 2011). This relation was also observed when chewing side preference in individuals was examined (Wilding, 1993). Direct measurement of worn facet areas on the arch occlusal surfaces revealed left-to-right area differences in approximately half of a sample of young adults, suggesting that about one individual in two had a preferred side of mastication (Bourdiol and Mioche, 2000). Dental wear differences are also found for both age and sex (Bourdiol et al., 2007). Increase in the number of mastication cycles was found when masticating on the side with fewer wear facets, suggesting a more difficult adaptation of the masticatory process (Bourdiol and Mioche, 2000).

Finally, dental abrasion is correlated with better oral health, fewer carious lesions due to dental plaque removal by an active mastication, Knychalska-Darman et al. (1972) and Taylor (1975) healthier periodontal tissues, Ainamo (1972) and good occlusal masticatory function. Masticatory function adapts to such slow, life-long gradual changes probably by plastic modifications of patterns in the central nervous system. The dental wear process, when normally progressive and evenly distributed along the dental arch, is thus physiological, at least for as long as the teeth and their supporting structures remain functional (Brace, 1977; Kaidonis, 2008).

## Adaptation of Mastication to Changes in Functional Occlusion

**FIGURE 3:**
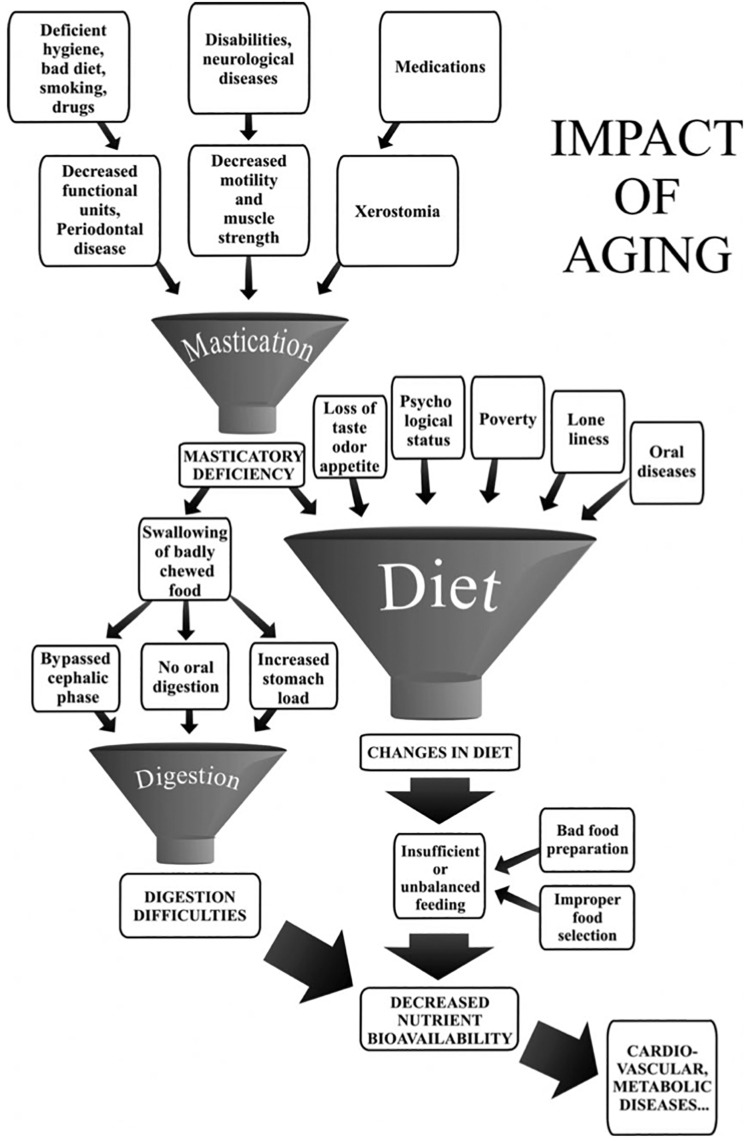
Impact of aging on mastication-dependent nutrient bioavailability. Impaired mastication may cause decreased nutrient bioavailability through changes in diet. The diagram emphasizes that mastication deficiency is only one of the many factors that may induce a change in the diet. Similarly, mastication deficiency or digestion difficulties may have several causative factors. The multiplicity of factors liable to lead to decreased nutrient bioavailability rules out any fully convincing demonstration of an etiological relationship between masticatory deficiency and decreased nutrient availability (taken from Peyron et al., 2017, with permission).

Here we consider malocclusion exclusively from a functional point of view. The question is to what extent malocclusion impacts chewing. It is claimed that malocclusion has little impact on chewing (Mohlin and Kurol, 2003). This appraisal is based on casual clinical situations but does not take full account of the broad variety of situations covered by the ill-defined term malocclusion. There is a huge difference between morphological malocclusion such as limited anterior dental crowding in which the disorder is purely esthetic, and major malocclusions resulting from maxillofacial oncological surgery or sudden severe cerebral palsy. All shades are possible between these two extremes. How does masticatory function adapt completely, partially or unsuccessfully to the various possibilities across this broad malocclusion range? Whether patients have mastication characterized by compensatory adaptation or by incapacity has important clinical consequences that have still not been satisfactorily addressed. Mastication training could be proposed when compensatory adaptation is possible, and special food provision or a modified diet should be advised in cases of incapacity. A related question is the direction of the change: most often, spontaneous evolution, either gradual or sudden, tends toward a worsening of the occlusion conditions of mastication; a gradual or sudden change for the better is expected after therapeutic interventions. Apart from saliva, which must be of adequate quality and quantity, two factors seem to affect the extent of adaptation: (i) number of functional teeth and (ii) masticatory forces (Hatch et al., 2000; Kosaka et al., 2018). The presence of enough functional interarch posterior tooth-to-tooth contacts as indicated by the PFU concept is a determining factor (van der Bilt, 2011; Tanaka and Shiga, 2018). Below are two examples of the role of PFUs among many others. Firstly, shortened dental arches, i.e., dental arches with no more than one PFU in the molar area, have been proposed as a therapeutic solution (van der Bilt, 2011). Persons with shortened dental arches tend to be satisfied with their oral function. They are, however, in a situation of compensatory adaptation, since they need twice as many strokes to obtain the same bolus as they would with complete dental arches (Fontijn-Tekamp et al., 2000). Secondly, Decerle et al. (2013) studied young adults with multiple carious lesions causing a decrease in the number of PFUs. Maximum adaptive capacity was reached; they evidenced an impaired masticatory capacity with a d50 well above the MNI value of 4 mm. The presence of a healthy neuromuscular system is also a determining factor controlling mastication through changes in muscle strength and coordination. In Down syndrome, the neuro-muscular defect negatively impacts mastication, independently of a smaller number of PFUs (Hennequin et al., 1999). The mastication deficiency induced by Parkinson disease (Ribeiro et al., 2016) becomes more marked as the disease progresses (Bakke et al., 2011). Very advanced age is another possible factor in neuromuscular system decline (Osterberg et al., 1996). However, several studies have shown that outside extreme vital decline, elderly subjects were able to adapt to the age-induced changes provided there were not too many interfering problems (Peyron et al., 2017), and particularly if they had a sufficient number of PFUs (Kohyama et al., 2003; Kosaka et al., 2018). However, the effect of age is often one factor among many, where the specific role of any single one is difficult to specify. A good illustration is given by the effect of aging on mastication, as shown in Figure 3.

The adaptation of mastication to occlusion can also be observed in the opposite direction, i.e., as adaptation not to a worsened occlusal state but to an upgraded one. Therapeutic rehabilitation offers opportunities to study this adaptation. Occlusal rehabilitation diversely improves mastication efficiency. This clearly depends on the method of rehabilitation. Complete dentures do not fully correct food comminution compared with a healthy dentate state. Longer mastication time and more masticatory cycles are necessary to obtain acceptable food bolus comminution. In the overwhelming majority of cases, complete denture wearers are unable to reach the performance of naturally dentate individuals (Helkimo et al., 1978; Wayler and Chauncey, 1983; Veyrune et al., 2007; van der Bilt, 2011). Many complete denture wearers are clearly in a state of mastication incapacity, while others manage a compensatory adaptation (Mishellany-Dutour et al., 2008). Again, compensatory adaptation or incapacity should be differentiated to offer appropriate therapeutic advice. The quality of prosthodontics is another factor. Some authors have described a strong improvement after complete denture renewal (Gunne, 1985; Berteretche et al., 2015) and others have reported more nuanced observations (Garrett et al., 1996; Veyrune et al., 2005). In every case, it seems that complete denture wearers have first to adapt to their new prosthetic device first to control their mastication behavior, and second to improve its result in terms of mastication efficiency (Yurkstas and Emerson, 1964; Fontijn-Tekamp et al., 2000; Veyrune et al., 2005). Up to 1 year has been reported as the time needed for maximal recovery of mastication after delivery of a new denture (Goiato et al., 2010). This long time might be shortened, and the recovery made more complete with a training program aimed at relearning a masticatory praxis adapted to the new dental prosthesis.

Removable partial dentures offer only a poor addition to efficient occlusal contacts. Even with removable partial dentures, the number of residual natural PFUs controls chewing. For most authors, improved comminution performance is obtained with removable partial dentures (Liang et al., 2015), but it never reaches normality (Bessadet et al., 2013; Liang et al., 2015; Tanaka and Shiga, 2018), with some authors describing no difference in comminution performance with or without removable partial dentures being worn (Liedberg et al., 1995). Subjective feelings of patients about mastication may, however, be improved by removable partial dentures (Gunne, 1985). Many parameters point to incomplete mastication with removable partial dentures. Mastication frequency remains low, a mark of abnormal mastication. The modest improvement brought about by removable partial dentures, and the dominant role of natural PFUs in mastication, is well demonstrated by no difference (Fueki et al., 2011) or only a slight difference (Liang et al., 2015) between mastication efficiency with shortened dental arch and with adjunction of a removable partial denture. The decreased number of natural PFUs also determines diminished biting forces (Helkimo et al., 1977; Ikebe et al., 2010). Subjects tend to compensate for decreased mastication efficiency by increasing the number of strokes, which is compensatory adaptation, and by selecting a soft diet (Liedberg et al., 2004). The exclusion of hard food and selection of soft diet can have a demonstrable (Inomata et al., 2015), a small impact (Wallace et al., 2018) or no impact (Liedberg et al., 2004) on nutrition and health. We can expect very different results depending on contrasting conditions. For example, the number and distribution of missing teeth, the type of food tested, and the tests used to assess the mastication deficiency/adaptation are powerful factors explaining differences in results. In summary, depending on the individual condition, mastication may be characterized by compensatory adaptation or by incapacity.

There is an unsubstantiated belief that implant-supported bridges allow complete recovery of masticatory function. Pioneer papers using pre/post-treatment design showed a clear improvement of mastication (Lindquist and Carlsson, 1985; Carlsson and Lindquist, 1994) especially when the initial oral state was so degraded that it needed major oral rehabilitation. Many of these pioneer papers also reported an adaptation process after these new conditions were set, with a progressive enhancement of mastication parameters in the subsequent months or years (Lindquist and Carlsson, 1985; Lundqvist and Haraldson, 1992; Akeel et al., 1993; Lundqvist, 1993). A strong subjective satisfaction with mastication accompanied the improvement in objective criteria (Lindquist and Carlsson, 1985; Lundqvist and Haraldson, 1992; Carlsson and Lindquist, 1994; Veyrune et al., 2013). However, comparison with healthy dentate subjects was seldom made (Carlsson and Lindquist, 1994). It was long after the initial pioneer period that a careful controlled study compared mastication function of subjects receiving implant-supported bridges with a healthy full dentate group (Grigoriadis et al., 2011). Implant-supported bridges involved one or both jaws. The evaluation was done at least 1 year after treatment. Electromyographic recording while chewing model foods with progressively increasing hardness revealed that jaw movements were affected in the group of subjects with dental implants. Unlike the control subjects, who increased muscle activity with hardness and decreased it near the end of the masticatory sequence with related changes in jaw movements, the participants with implants used similar muscle activities and jaw movements irrespective of both food type and time in the masticatory sequence. For the authors, this lack of adaptation probably relates to a lack of neural control due to the absence of periodontal receptors (see review in Avivi-Arber and Sessle, 2018). Implant-retained overdentures also strongly ameliorated masticatory efficiency and patient satisfaction, which remained high for a long time (Awad et al., 2000; Bakke et al., 2002; van der Bilt et al., 2012). Mastication parameters were observed to approach the values obtained in normal dentate subjects (Haraldson et al., 1988; Heckmann et al., 2009). This also applies to different type of implant-retained overdentures (Tang et al., 1999; Feine et al., 2002; Awad et al., 2003; van der Bilt et al., 2012), including mini-implants (Batisse et al., 2016; Goiato et al., 2018; Yao et al., 2018). The improvement with implant-retained overdentures was delayed for more than 6 months after rehabilitation treatment. This is because deeply anchored chewing habits corresponding to the previous oral conditions may be maintained despite conditions that are more favorable (Garrett et al., 1998; van der Bilt et al., 2010a; Okoński et al., 2011; Batisse et al., 2016). Maladaptive neuroplasticity may explain deeply anchored chewing habits (Avivi-Arber and Sessle, 2018), and positive plasticity might have been enhanced by a training program (Kumar et al., 2018).

**FIGURE 4:**
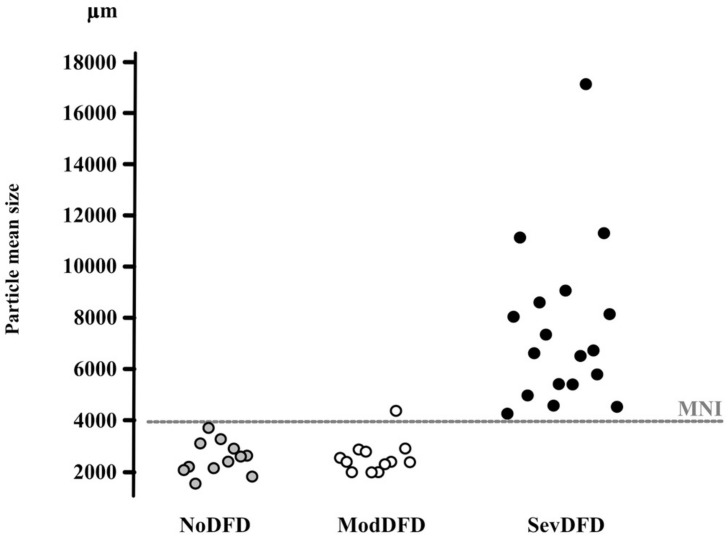
Correspondence between median particle size (d50) values and orthodontic/orthognathic treatment needs. d50 values (μm) of individuals in three groups of adults are given on the *y*-axis. NoDFD (no treatment needed), ModDFD (indication for orthodontic treatment alone), SevDFD (indication for surgical treatment). The horizontal dashed line corresponds to the Masticatory Normative Index (MNI). Individual subject values are positioned on the *x*-axis according to their corresponding group to facilitate reading (taken from Bourdiol et al., 2017, with permission).

Subjects with dento-facial deformities and orthodontic needs may present a deficient masticatory function (Tate et al., 1994; van den Braber et al., 2001; English et al., 2002; Iwase et al., 2006; N’Gom et al., 2007; Magãlhaes et al., 2010; Picinato-Pirola et al., 2012; Abrahamsson et al., 2013; Hennequin et al., 2015). These subjects often display an interarch discrepancy or inadequacy, as reflected by the significantly reduced functional area (Kobayashi et al., 1993; Henrikson et al., 1998; Magãlhaes et al., 2010; Bourdiol et al., 2017). However, dento-facial deformities may lead to two quite different endpoints: satisfactory masticatory function through successful compensatory adaptation, or strongly impaired mastication because adaptation proves impossible (incapacity) (Mishellany et al., 2006; Woda et al., 2011). The relation between severity of dento-facial deformities and extent of mastication deficiency has been assessed (Bourdiol et al., 2017) (Figure 4). Subjects needing only orthodontic treatment, i.e., presenting with a moderate dento-facial deformity, succeeded in making a normal food bolus. They might have a lowered masticatory efficiency as indicated by food bolus particle size measured after a limited number of cycles. It can be inferred that they adapted their masticatory function, mostly by increasing the number of chewing cycles and the duration of the masticatory sequence. Members of this group thus achieved a normal functional result. By contrast, subjects confronted with severe dento-facial deformities and needing combined orthodontics and surgery, failed to adapt. They swallowed insufficiently prepared food or selected their diet. Both orthodontic treatment alone (Henrikson et al., 2009) and orthodontics associated with orthognathic surgery (Shiratsuchi et al., 1991; Kikuta et al., 1994; Zarrinkelk et al., 1995; van der Braber et al., 2005) improved masticatory performance, at least partially. It must be noted that after orthognathic surgery, improvement of masticatory efficiency does not occur immediately. The functional benefit of a combined orthodontic and surgical approach appears progressively in the course of at least 1 year (Kikuta et al., 1994; Iwase et al., 2006; Magãlhaes et al., 2010), but may never reach complete normality (van der Braber et al., 2006). The delayed recovery is probably due, at least in part, to the time needed to learn a new masticatory praxis enabling the patient to master the new anatomic conditions.

Rehabilitating intervention in patients with Down syndrome led to an increased number of PFUs. This increase improved chewing, with a decreased occurrence of food rejections, and a smaller median size of bolus particles and fewer masticatory cycles before bolus swallowing (Hennequin et al., 2015).

**FIGURE 5:**
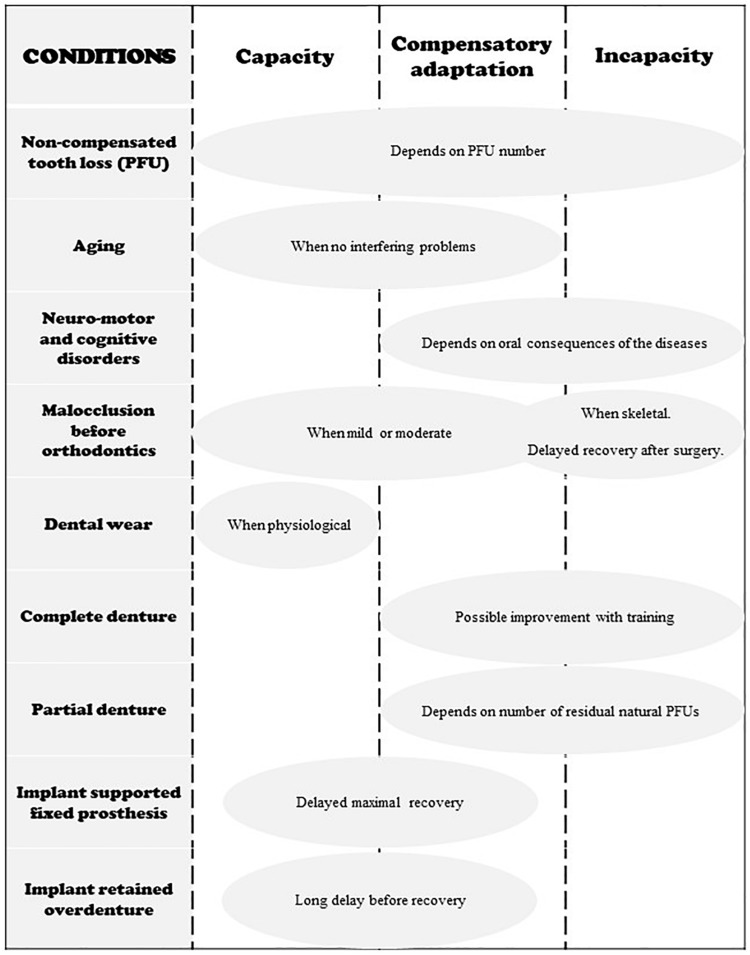
Schematic representation of the three states of adaptation in regard to different occlusal conditions. Neuromotor and cognitive disorders include Down Syndrome, stroke, Parkinson disease and other neuro-degenerative disorders, facial damage, mental illness, etc.

The conditions that govern capacity, compensatory adaptation and incapacity are summarized in Figure 5.

## Effect of Adaptation of Mastication on Nutrition

One component of adaptation is behavioral adaptation through diet choice. When in a state of incapacity, subjects presenting with deficient mastication may exclude hard-to-chew food from their diet. For example, removable denture wearers reject hard foods and restrict themselves to those easy to chew (Liedberg et al., 2005). Others may shorten chewing time and swallow a coarse food bolus. In both cases, there may be negative health consequences, mostly on digestive function and nutrition as described below (see Peyron et al., 2017). There is overwhelming evidence of a correlation between masticatory deficiency and malnutrition (N’Gom and Woda, 2002; Hutton et al., 2002). However, a causal relationship remains to be demonstrated (Mioche et al., 2004; Rémond et al., 2015). Based on a systematic review with 11 studies using a multivariate approach, van Lancker et al. (2012) supported an independent association between oral health status and malnutrition. Changes in diet depended on masticatory function according to number of teeth. However, little modification was seen in nutrient concentrations in blood (Sheiham et al., 2001). A causal relationship between masticatory function and blood availability of nutrients was shown in a trial design comparing full dentures with and without supporting implants (Morais et al., 2003). Further research is needed to seek evidence for a causal relationship between mastication, oral health and malnutrition. Several points, however, must be clarified for this approach. The common assumption that the oral stage of eating is a minor function gainsays the obvious vital role of food. Eating is so important that evolution has engineered overlapping functions shared by several segments of the upper digestive tract. In this way, failure of any one organ will not mean starvation. Mouth and stomach may thus be considered as performing multiple mechanical activities with built-in functional redundancy. Hence mouth function may have been underestimated.

## Conclusion

Increasing knowledge about the processes by which mastication adapts in response to food properties or oral status is challenging for researchers, clinicians and patients. This review describes the different adaptation processes for mastication that enable individuals to maintain healthy nutritional status. Any modification of dental status, saliva flow or neuromuscular apparatus can affect mastication and nutrition. Oral incapacities affect mastication for solid and semi-solid foods. Eating liquid foods or purées can facilitate deglutition but this bypasses the triggering of the cephalic phase and alters the digestive process. Dental professionals need to be alert to these concepts and to mastication evaluation.

## Author Contributions

PB, MH, M-AP, and AW contributed to the concepts and bibliographic analysis. AW and PB made first writing which was later corrected by the four authors.

## Conflict of Interest

The authors declare that the research was conducted in the absence of any commercial or financial relationships that could be construed as a potential conflict of interest.
